# Dr. John A. Larrabee

**Published:** 1898-07

**Authors:** 


### OBITUARY.

---

Dr. John A. Larrabee, of Louisville, Ky., one of that city's best-known physicians and one also who was well known in his profession all over the country, died June 12, 1898, of Bright's disease, at the age of fifty-eight. He was a native of Maine and a man of wide popularity. He contributed largely to medical journals and was the author of many treatises on diseases of children and was a teacher of ability. He held the chair of obstetrics and diseases of children at the Hospital College of Medicine and was president of the joint faculties of the medical and dental departments of Central University of Kentucky. Appropriate resolutions of respect were adopted by these bodies in joint session and they attended the funeral in a group.

